# Study protocol: a multicenter, uncontrolled, open-label study of palivizumab in neonates, infants, and preschool children at high risk of severe respiratory syncytial virus infection

**DOI:** 10.1186/s12887-021-02567-6

**Published:** 2021-03-02

**Authors:** Masaaki Mori, Shinichi Watabe, Tomoaki Taguchi, Hisaya Hasegawa, Mika Ishige, Naoyuki Tanuma, Akihiro Hirakawa, Ryuji Koike, Satoshi Kusuda

**Affiliations:** 1 Department of Pediatrics, Tokyo Medical and Dental University Medical Hospital, 1-5-45 Yushima, Bunkyo-ku, Tokyo, 113-8519 Japan; 2 Department of Pediatrics, Kurashiki Central Hospital, 1-1-1 Miwa, Kurashiki, Okayama, 710-8602 Japan; 3 Department of Pediatric Surgery, Kyushu University Hospital, 3-1-1 Maidashi, Higashi-ku, Fukuoka, 812-8582 Japan; 4 present affiliation: Fukuoka College of Health Science, 2-15-1 Tamura, Sawara-ku, Fukuoka, 814-0193 Japan; 5 Division of Neonatal Intensive Care, Tokyo Women’s Medical University Medical Center East, 2-1-10 Nishiogu, Arakawa-ku, Tokyo, 116-8567 Japan; 6 Department of Pediatrics and Child Health, Nihon University School of Medicine, 1-6 Kanda Surugadai, Chiyoda-ku, Tokyo, 101-8309 Japan; 7 Department of Pediatrics, Tokyo Metropolitan Fuchu Medical Center for the Disabled, 2-9-2 Musashidai, Fuchu, Tokyo, 183-8553 Japan; 8 Clinical Research Center, Tokyo Medical and Dental University Medical Hospital, 1-5-45 Yushima, Bunkyo-ku, Tokyo, 113-8519 Japan; 9 Department of Pediatrics, Faculty of Medicine, Kyorin University, 6-20-2 Shinkawa, Mitaka, Tokyo, 181-8611 Japan

**Keywords:** Airway stenosis, Congenital esophageal atresia, Efficacy, Inherited metabolic disease, Neuromuscular disease, Palivizumab, Pediatric patient, Pulmonary hypoplasia, Respiratory syncytial virus infection

## Abstract

**Background:**

The prophylactic use of anti-respiratory syncytial virus (RSV) antibody (palivizumab) for severe RSV infection is not approved in Japan in specified groups of infants with neuromuscular diseases or other rare diseases associated with reduced ventilation competence or difficulty in expectoration, which increase the risk of exacerbation of severe RSV infection. The objective of this study is to investigate the efficacy, safety, and pharmacokinetics of palivizumab in pediatric patients with those rare diseases for which palivizumab is not indicated at present.

**Methods/design:**

This study is a multicenter, uncontrolled, open-label study planned to be carried out between July 1, 2019 and June 30, 2022 at 7 medical institutions in Japan. The study population will be recruited from among neonates, infants, or children aged 24 months or younger with a condition falling under any of the following 5 disease groups: pulmonary hypoplasia, airway stenosis, congenital esophageal atresia, inherited metabolic disease, or neuromuscular disease. The planned sample size is 18 subjects, including at least 3 subjects per disease group. Throughout the RSV season, at least 4 continuous doses of palivizumab will be administered intramuscularly at 15 mg/kg at intervals of 30 days. The efficacy and safety of palivizumab will be comprehensively evaluated based on the incidence of RSV-related hospitalization, and serum palivizumab concentration, serum anti-palivizumab antibody concentration, and the occurrence of adverse events/reactions after the start of palivizumab treatment.

**Discussion:**

This study will evaluate the efficacy and safety of palivizumab in pediatric patients with rare diseases which place them at high risk of severe RSV infection, but which fall outside the current indications for palivizumab prophylaxis. The generated data will have implications for the regulatory approval of prophylactic palivizumab treatment in this patient group.

**Trial registration:**

This study has been prospectively registered in Japic Clinical Trials Information, which is managed and administered by the Japan Pharmaceutical Information Center (registration number: JapicCTI-194946, registration date: September 10, 2019).

## Background

Respiratory syncytial virus (RSV) is responsible for approximately 50% of pneumonia cases and 50 to 90% of bronchiolitis cases in infants and represents the leading cause of infection-related hospitalization of infants [1]. Currently, no vaccine to prevent or drug to treat RSV infection is available. Hence, from the viewpoint of disease control, priority is given to preventing aggravation of the disease.

Palivizumab, a specific human monoclonal antibody, is currently the only approved agent to prevent aggravation of RSV infection. It is manufactured and marketed by AbbVie GK. Palivizumab was discovered by MedImmune LLC (USA) [2] and approved for the indication “Prevention of severe lower respiratory tract diseases caused by RSV infection in pediatric patients at high risk of RSV infection” in June 1998 in the USA and later in European countries [3]. Palivizumab has been used in at least 70 major countries, including countries in Europe and North America [4].

A bridging study conducted in Japan verified the validity of extrapolating overseas efficacy data to Japan [5]. After reviewing international and domestic clinical trials, the Ministry of Health, Labor, and Welfare of Japan approved preventive treatment with palivizumab for children. The use of palivizumab for neonates and infants aged ≤12 months who were born prematurely at ≤28 weeks gestational age, aged ≤6 months who were born prematurely at 29–35 weeks gestational age or aged ≤24 months associated who have treated for bronchopulmonary dysplasia (BPD) within the past 6 months, congenital heart disease (CHD), immunodeficiency and Down’s syndrome [6].

To better understand the prevalence of RSV infections in Japan, Mori et al. conducted nationwide questionnaire surveys which identified immunodeficiency, Down syndrome, chromosomal abnormalities, and neuromuscular diseases as important underlying diseases associated with severe RSV infection [7, 8]. On the basis of these findings, a written request for the use of unapproved drugs/off-label use of approved drugs was submitted by the Pediatric Rheumatology Association of Japan and the Japanese Society of Pediatric Hematology/Oncology. In accordance with the “Request for the Development of Unapproved Drugs/Off-label Use of Approved Drugs”, a clinical study was conducted to expand the indication of palivizumab to include neonates, infants, and preschool children with immunodeficiency or Down syndrome [9]. These additional indications were approved in Japan in August 2013.

However, of the underlying diseases which were shown to be associated with severe RSV infection in the above surveys [7, 8], neuromuscular diseases were not included in the indications additionally approved in 2013. Given this, the Japan Society of Perinatal and Neonatal Medicine and other organizations requested various relevant academic societies (the Japanese Society for Inherited Metabolic Diseases, the Japanese Society of Child Neurology, the Japanese Society of Pediatric Pulmonology, and the Japanese Society of Pediatric Surgeons) to provide opinions on relevant underlying diseases for which palivizumab is currently not approved. As a result, it was concluded that the indications of palivizumab should be expanded to include pediatric patients with diseases associated with reduced ventilation competence or difficulty in expectoration. This includes the following 5 disease groups: pulmonary hypoplasia, airway stenosis, congenital esophageal atresia, inherited metabolic disease, and neuromuscular disease. The mechanisms for severe RSV infection in these disease groups are presented in Table 1. All of these diseases are associated with a high risk of severe RSV infection, or render children vulnerable and thereby make it difficult to treat aggravated RSV infections. To date, however, information about the use of palivizumab in pediatric patients with any of these underlying diseases has not been available.

**Table 1:** Mechanisms of severe respiratory syncytial virus (RSV) infection in the rare diseases targeted in this study

Disease group	Mechanism for severe RSV infection	
	Reduced ventilation competence^a^	Difficulty in expectoration^b^
Pulmonary hypoplasia		○
Airway stenosis	○	○
Congenital esophageal atresia	Δ	○
Inherited metabolic disease	○	
Neuromuscular disease		○

○ = Yes, Δ = Yes, depending on the condition, Blank = not applicable

^a^Reduced ventilation competence: Because of their reduced ventilation competence, children with airway stenosis who develop RSV-induced lower respiratory tract infection may experience hypoxia

^b^Difficulty in expectoration: Because of the difficulty in expectoration, patients cannot eliminate RSV via the sputum, which results in prolonged infection. Secretions retaining in the respiratory tract make ventilation difficult

Therefore, the objective of this study is to investigate the efficacy and safety of palivizumab in pediatric patients falling under each of the above 5 disease groups. The findings of this study will have implications for the regulatory approval of palivizumab use in these pediatric patients.

## Methods/design

### Aims and setting

The objective of this study is to investigate the efficacy, safety, and pharmacokinetics of palivizumab in pediatric patients with a disease classified under any of the following 5 disease groups associated with reduced ventilation competence or difficulty in expectoration, which place patients at a high risk of exacerbation of (severe) RSV infection and for which palivizumab is not indicated at present, namely pulmonary hypoplasia, airway stenosis, congenital esophageal atresia, inherited metabolic disease, and neuromuscular disease. The efficacy and safety of palivizumab will be comprehensively evaluated. The study period is set from July 1, 2019 to June 30, 2022. The enrollment period is planned from July 1, 2019 to December 31, 2020. The study will be conducted 7 medical institutions sites in Japan.

### Participant recruitment and determination of sample size

The study population will be recruited from among neonates, infants, or preschool children who are 24 months or younger with any of the following conditions: pulmonary hypoplasia (congenital diaphragmatic hernia and congenital cystic lung disease), airway stenosis (pharyngeal stenosis, laryngeal stenosis, airway/bronchial stenosis, and airway/bronchial malacia), congenital esophageal atresia, inherited metabolic disease (diseases affecting gluconeogenesis or the glycolytic energy production pathway (e.g., fatty acid oxidation disorders and carnitine cycle disorders), diseases associated with mitochondrial abnormalities (e.g., mitochondrial respiratory chain disorders), diseases affecting the metabolism of amino acids or proteins (e.g., organic acid metabolism disorders and urea cycle disorders, lysosomal diseases, peroxisomal diseases, etc.), as well as neuromuscular diseases (muscular dystrophy, congenital myopathy, spinal muscular atrophy, myotonic dystrophy, and myasthenic syndrome). Estimated yearly numbers of pediatric patients falling within these disease groups in Japan are shown in Table 2.

**Table 2:** Rare diseases targeted in this study, their component individual diseases and estimated number of pediatric patients in Japan

Disease group	Representative individual diseases	Estimated number of patients in Japan per year (age 0 to 24 months)
Pulmonary hypoplasia	Congenital diaphragmatic hernia Congenital cystic lung disease	~ 1000
Airway stenosis	Pharyngeal stenosis Laryngeal stenosis Airway/bronchial stenosis Airway/bronchial malacia	~ 500
Congenital esophageal atresia	Congenital esophageal atresia	~ 200
Inherited metabolic disease	Diseases affecting gluconeogenesis or the glycolytic energy production pathway (e.g., fatty acid oxidation disorders and carnitine cycle disorders) Diseases associated with mitochondrial abnormalities (e.g., mitochondrial respiratory chain disorders) Diseases affecting the metabolism of amino acids or proteins (e.g., organic acid metabolism disorders and urea cycle disorders) Lysosomal diseases Peroxisomal diseases	250 to 300
Neuromuscular disease	- Muscular dystrophy - Congenital myopathy - Spinal muscular atrophy - Myotonic dystrophy - Myasthenic syndrome	~ 300

The planned sample size is 18 subjects, with at least 3 subjects per disease group planned to be enrolled in the study. As for the rationale for this sample size, this study targets pediatric patients with very rare diseases which are associated with high risk of severe RSV infection, as requested by relevant pediatric medical associations. In general, the RSV epidemic season starts in July and ends the following March. For the purpose of this study, subjects need to start treatment with palivizumab in the early stage of an RSV season and continue the treatment for at least 4 months during the season. This means that subjects can be recruited only in several months, and not throughout the entire year. Given these restrictions, a sample size of at least 3 per disease group (at least 15 subjects in the full analysis set) was considered sufficient to determine the incidence of RSV-related hospitalization, which is the primary efficacy endpoint of the study, and the blood concentrations of palivizumab. Assuming a dropout rate of 20%, the planned sample size of 18 was determined.

### Inclusion criteria

The study will include patients who meet all of the following criteria: (1) a legally acceptable representative of the child has provided written informed consent to the protocol treatment and examinations in the study. (2) The child is 24 months or younger at the time the consent is provided. (3) At the time the consent is provided, the child has a disease classified into any of the following disease groups, which are characteristically associated with reduced ventilation competence or difficulty in expectoration if infected, thereby resulting in exacerbation of the infection, and which requires prophylactic treatment with palivizumab as determined by the investigator or subinvestigator: pulmonary hypoplasia, airway stenosis, congenital esophageal atresia, inherited metabolic disease, and neuromuscular disease. (4) At the time the consent is provided, the child is being managed continuously on an outpatient basis.

### Exclusion criteria

This study will exclude patients meeting any of the following criteria: (1) At baseline, the child meets any of the following conditions for which the use of palivizumab is already approved: 1) the child is a neonate or infant aged 12 months or younger born prematurely at 28 weeks or less of gestation. 2) The child is a neonate or infant of 6 months or younger born prematurely at 29 to 35 weeks of gestation. 3) The child is a neonate, infant, or preschool child of 24 months or younger treated for bronchopulmonary dysplasia within the past 6 months. 4) The child is a neonate, infant, or preschool child of 24 months or younger treated for congenital heart disease with hemodynamic abnormality. 5) The child is a neonate, infant, or preschool child of 24 months or younger with immunodeficiency. 6) The child is a neonate, infant, or preschool child of 24 months or younger with Down syndrome. (2) The child has previously received palivizumab. (3) The child has received another investigational product within 3 months before the first dose of palivizumab in this study or has received another investigational product within a period of less than 5 times the elimination half-life of this product in blood. (4) The child has had an active infection, including RSV infection, during the screening period. (5) The child has required oxygen inhalation, artificial ventilation, membrane oxygenator, continuous positive airway pressure, or other assisted ventilation during the screening period. However, a child who has been receiving oxygen inhalation at a stable setting for the treatment of an underlying disease for 2 weeks or longer may be enrolled in the study. (6) The child has any serious complication (e.g., hepatic failure, tonic convulsion) other than immunodeficiency and renal failure. (7) The child has a history of apnea. (8) The child has a history of hypersensitivity to any component of the investigational product. (9) The child has a history of any serious adverse drug reaction or allergy to an immunoglobulin preparation(s). The child has a history of hypersensitivity to immunoglobulin, blood, or other protein preparations. (10) The child is not expected to survive for at least 1 year after the first dose of palivizumab. (11) The child is ineligible for participation in this study for any other reason(s) in the investigator’s or subinvestigator’s opinion.

### Withdrawal criteria

Criteria for withdrawal of individual subjects from study treatment are as follows: (1) The subject dies. (2) Continuation of the study is inappropriate because of an adverse event(s) in the investigator’s or subinvestigator’s opinion. (3) Continuation of the study is inappropriate because of poor response in the investigator’s or subinvestigator’s opinion. (4) Continuation of the study is inappropriate for any other reason(s) in the investigator’s or subinvestigator’s opinion. (5) The subject’s legally acceptable representative does not want the subject to continue participation in the study and the subject has received 3 or fewer doses of palivizumab at the time of that decision. (6) Withdrawal from the study has been decided for any reason(s) other than (1) to (5).

### Intervention

Investigational product: palivizumab. Throughout the RSV season, subjects will receive at least 4 continuous doses of palivizumab (genetical recombination), administered intramuscularly at 15 mg/kg at intervals of 30 days. If the injection volume exceeds 1 mL, the injection solution must be given in divided doses. Subjects are allowed to receive more than 4 continuous doses (up to 9 doses) of palivizumab during the same RSV season, if such doses are requested by their legally acceptable representatives and approved by the investigator or subinvestigator. The above dose and administration method were selected with reference to the dosage and administration recommended in the package insert of palivizumab.

### Observations/examinations/evaluations and study schedule

The study will be divided into a screening period (28 days), a study period (181 days), and a follow-up period after the last dose of palivizumab (30 days). During the screening period, candidate subjects will be assessed for their baseline characteristics, eligibility, medical history and concurrent diseases, concomitant drugs/therapies, vital signs, and height. A medical examination/interview and an RSV infection test will be performed, and their informed consent will be obtained.

At each of the following visits, the subjects will receive palivizumab treatment after careful evaluation by the investigating physician. Assessment of concomitant drugs/therapies, conduction of a medical examination/interview, RSV infection test, measurement of vital signs, height, body weight, as well as the performance of laboratory tests (blood/urine collection), blood collection for blood drug concentration measurement, and blood collection for anti-palivizumab antibody assay will be carried out as detailed in the study schedule presented in Table 3. Investigation and recording of adverse events and adverse reactions will be carried out continuously from the first visit throughout to the end of the study. Adverse reactions will be followed up until they are resolved.

**Table 3:**
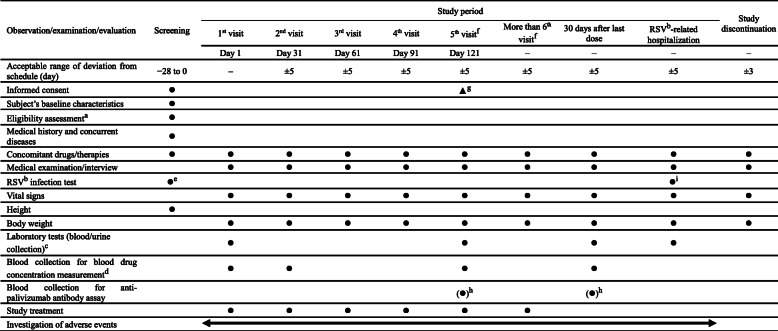
Study schedule

^a^The eligibility of each subject will be assessed during the screening period

^b^RSV, respiratory syncytial virus

^c^Hematology, blood biochemistry, and urinalysis

^d^Blood collection for blood drug concentration measurement must be performed prior to administration of palivizumab

^e^For a RSV infection test during the screening period, a RSV rapid test (immunochromatography) kit will be used

^f^When the 5th, 6th, or 7th dose of palivizumab is administered, efficacy and safety measurements must be performed on Day 151, 181, or 211, respectively

^g^The legally acceptable representative of the subject who requests continuation of treatment with palivizumab must provide written consent to the continued treatment prior to the administration of palivizumab at the 5th visit

^h^First, the subject’s health condition must be checked. Blood collection for laboratory tests should come first, followed by blood collection for anti-palivizumab antibody assay if possible

^i^ RSV infection upon RSV-related hospitalization will be tested using the polymerase chain reaction (PCR) method

### Main outcome measurements and endpoints

The outcome of the study will be comprehensively evaluated based on results for the following endpoints: efficacy, safety, and pharmacokinetics.

#### Efficacy

The primary endpoint will be the percentage of subjects requiring RSV-related hospitalization between the first dose of palivizumab and 30 days after the last dose. Secondary endpoints will be: the percentage of subjects requiring oxygen inhalation, artificial ventilation, membrane oxygenator, continuous positive airway pressure, other assisted ventilation, or management in an intensive care unit owing to RSV infection between the first dose of palivizumab and 30 days after the last dose.

#### Safety

Adverse events and adverse reactions will be summarized by grade, seriousness, and timing of onset using the number of subjects with events/reactions, and the incidence and number of events/reactions. An adverse event is defined as any unfavorable or unintended sign, symptom, disease, or an abnormal change in laboratory test parameters (hematology, blood biochemistry, and urinalysis), vital signs, or body weight occurring between the first dose of palivizumab and 30 days after the last dose or early study discontinuation. Events (signs, symptoms, and diseases) present prior to the first dose of palivizumab should be reported as adverse events if they are aggravated after the start of the study treatment compared with baseline and meet the conditions in “*Assessment of adverse events*” below.

If any adverse event is found in a subject on medical examination, the investigator or subinvestigator will provide appropriate interventions and take all necessary measures. The investigator or subinvestigator will determine whether a change observed in a subject is a clinically significant change (abnormal change) according to the following criteria based on the necessity of a medical intervention. Abnormal changes will be reported as adverse events. 1) The change in the test parameter requires the withdrawal of the subject from the study treatment. 2) The change in the test parameter requires any drug or medical intervention for treatment. 3) The change in the test parameter requires any surgical intervention. 4) The change in the test parameter is otherwise of special medical interest in the investigator’s or subinvestigator’s opinion.

##### Assessment of adverse events

In this study, adverse events will be categorized and rated for severity according to the JCOG version of the Japanese translation of the Common Terminology Criteria for Adverse Events v5.0 (CTCAE v5.0/JCOG). In assessing the severity of each event, a grade which most accurately describes the severity of the event will be selected from Grades 1 to 5 according to their definitions. If the definition of a grade contains a specific intervention for the event, its grade will be determined based on the clinical necessity of that intervention. When the CTCAE and its Japanese version are revised in association with revision of the Medical Dictionary for Regulatory Activities (MedDRA) and its Japanese version MedDRA (MedDRA/J), safety will be evaluated using a combination of the corresponding latest CTCAE and MedDRA/J.

In this study, concurrent diseases observed during the screening period will be reported as adverse events when they are aggravated by at least 1 grade as determined according to CTCAE v5.0. Laboratory test abnormalities will also be rated for severity in the same manner. For each adverse event occurring in a subject, the investigator or subinvestigator will document the name, date of onset, intervention taken, date of resolution (date of last observation), grade, seriousness, outcome, and causal relationship in the case report form (CRF).

##### Follow-up investigation of adverse events

Adverse events will be followed up by the investigator or subinvestigator as necessary. Adverse events in untreated subjects will not be followed up. Adverse events that have not resolved by 30 days after the last dose of palivizumab will be followed up until their follow-up is considered medically unnecessary by the investigator or subinvestigator. Adverse reactions must be followed up until they resolve. The investigator or subinvestigator must report follow-up results of adverse events to the coordinating investigator. If follow-up of an adverse event is not performed, the reason must be provided in the CRF. The follow-up result of each event should be recorded in the CRF roughly between 30 days after the last dose of palivizumab and 4 weeks thereafter.

The above rules may not be applied if symptoms become chronically associated with the aggravation of the underlying disease or concurrent diseases or if it is difficult to continue observation of a subject because of the subject’s transfer to another hospital or for any other reason.

#### Pharmacokinetics

##### Serum palivizumab drug concentrations

Serum palivizumab concentrations (trough values) 30 days after the first, fourth, and last doses of palivizumab will be measured by AbbVie GK. Venous blood will be collected from each subject using a blood collection tube containing no separating agent. A sufficient amount of blood will be collected so that 0.8 mL of serum can be obtained at each blood collection. Samples are allowed to stand for 30 to 60 min and are centrifuged to separate serum. Then, 0.8 mL of the serum obtained is pipetted into 2 polypropylene vials with a screw cap (approximately 0.4 mL of aliquot per vial) using a plastic pipette. One of the 2 aliquots will be used as a spare sample. A label carrying the investigational product code, protocol No., subject ID, time point of blood collection, and date of blood collection will be attached to each vial. The obtained serum samples will be frozen at − 80 °C or lower within 2 h after blood collection and stored at the study site until collection by Tokyo Medical and Dental University.

### Additional outcome measurements

An additional outcome measurement will be serum anti-palivizumab antibody concentration.

Serum anti-palivizumab antibody concentrations will be measured by AbbVie GK. The investigator or subinvestigator will collect blood from the subject for measurement of anti-palivizumab antibody concentration 30 days after the fourth dose (before the fifth dose) and 30 days after the last dose. In subjects who have received 4 doses of palivizumab with the fourth dose as the last dose, the total number of blood collections is 1. If blood collection is scheduled for the same day as the study treatment, blood should be collected before administration of palivizumab. If scheduled blood collection cannot be performed before administration of palivizumab, it should be reported as a protocol deviation, but blood collection is still required. It should be confirmed that blood collection was performed before administration of palivizumab. The date of blood collection and the confirmation will be documented in the CRF. Blood samples will be collected and stored at each study site according to the procedures written in “Serum palivizumab drug concentrations” above.

### Completion, discontinuation, or suspension of the study (all or some of the study sites, and individual subjects)

The study will be completed when all subjects complete the study period, followed by submission of all CRFs and data lock. The coordinating investigator will notify persons who conduct the study, as well as the subinvestigators, data center staff, Data and Safety Monitoring Committee members, and other study personnel of the completion of the study.

### Statistical analysis

Data sets to be analyzed are the safety analysis set (consisting of all enrolled subjects who have received at least 1 dose of palivizumab), the full analysis set (same as the safety analysis set), and the per protocol set (consisting of subjects in the full analysis set who meet the following conditions and comply with the protocol). (1) Subjects who required no RSV-related hospitalization between the first dose of palivizumab and 30 days after the last dose and received at least 4 doses of palivizumab. (2) Subjects who required RSV-related hospitalization between the first dose of palivizumab and 30 days after the last dose and underwent observations/examinations between 5 days before and after hospitalization.

### Efficacy analysis

For the primary endpoint, the number and percentage (%) of subjects requiring RSV-related hospitalization between the first dose of palivizumab and 30 days after the last dose will be calculated with 95% Clopper-Pearson confidence intervals (CIs). The polymerase chain reaction method will be used to confirm RSV infection. The primary efficacy evaluation will be based on descriptive statistics without statistical hypothesis testing. For the secondary endpoints, the number and percentage (%) of subjects requiring oxygen inhalation, artificial ventilation, membrane oxygenation, continuous positive airway pressure, other assisted ventilation, or management in an intensive care unit owing to RSV infection between the first dose of palivizumab and 30 days after the last dose will be calculated with 95% Clopper-Pearson CIs. The period required for each of these interventions will also be summarized using mean, standard deviation, 95% CI, median, and range.

### Safety analysis

Adverse events and adverse reactions will be summarized using the number of subjects with events/reactions, and the incidence and number of events/reactions by grade, seriousness, and timing of onset. Adverse event terms reported in the CRFs by the investigator or subinvestigator will be translated to their corresponding lower level terms of the MedDRA. Adverse events will be summarized by system organ class and preferred term using the number of subjects with events, and the incidence and number of events.

Laboratory test parameters, vital signs, and body weight will be summarized for each time point using descriptive statistics. Data of each parameter will also be assessed to detect abnormal changes.

### Analysis of blood drug concentrations

Serum palivizumab concentrations will be summarized for each sampling point using descriptive statistics (number of observations, number of non-missing observations, arithmetic mean, median, standard deviation, coefficient of variance, minimum value, and maximum value). The 95% CI of the serum palivizumab concentration at each sampling point will also be calculated.

## Discussion

This study will assess the efficacy and safety of palivizumab in a specified group of pediatric patients with extremely rare disorders which put them at higher risk for severe RSV infection, but for which palivizumab is not indicated at present in Japan.

RSV infection is a common infection of the respiratory tract with the primary infection prevalent in children up to 2 years of age. In children with underlying diseases, immunocompromised children and preterm infants, RSV infection can become severe, necessitate hospitalization, and sometimes result in death [1].

Palivizumab, a humanized monoclonal antibody for the prevention of RSV infection, has been available in over 70 countries [3, 4]. In Japan, palivizumab has been approved for prophylactic use in infants at high risk for RSV infection, including preterm infants, immunocompromised infants, children with congenital heart disease and children with Down syndrome [5, 6]. However, the current indications do not cover all conditions shown to predispose infants to severe RSV infection.

In nationwide surveys in Japan which investigated the outcomes of RSV infections that occurred between August 2006 and July 2008, Mori et al. found that patients with neuromuscular disorders accounted for 16.5% of the children with underlying disease with poorer outcomes than other patient groups, representing an important patient group in which RSV prophylaxis would be indicated [7, 8]. Further, in American children who received at least one injection of palivizumab during any RSV season from 2002 to 2004, RSV-related hospitalization rates were significantly higher in children with congenital airway abnormality or severe neuromuscular disorders [10]. Moreover, in a prospective multicenter study in Germany covering 6 consecutive RSV seasons between 1999 and 2005, children hospitalized with RSV infection and neuromuscular impairment had a greater risk of requiring mechanical ventilation and developing seizures [11].

It is therefore highly desirable to expand the current indications for prophylactic palivizumab treatment to include all vulnerable pediatric patients in Japan. Since no data are available in Japan on the use of palivizumab in infants with the target diseases indicated in this study, the findings of this study will have implications for the regulatory approval of prophylactic palivizumab use in these pediatric patients.

Although the planned sample size of 18 patients in this study is small, it was considered sufficient to determine the incidence of RSV-related hospitalizations, which is the primary efficacy endpoint of the study, and the blood concentration of palivizumab. A possible limitation of the planned study might be the lack of a control/placebo group. If needed, RSV-related hospitalization rates from subjects who received palivizumab in this study will be compared to retrospectively available data from age-matched pediatric patients from the same disease groups who did not receive palivizumab.

In summary, the findings of this study will have implications for the expansion of current palivizumab treatment indications to include pediatric patients with pulmonary hypoplasia, airway stenosis, congenital esophageal atresia, inherited metabolic disease, and neuromuscular disease, with the aim of reducing the incidence of RSV-related hospitalizations and preventing severe RSV-related complications in vulnerable infants, ultimately improving quality of life.

## Data Availability

Not applicable.

## Abbreviations

CI: Confidence interval
CRF: Case report form
CTCAE v5.0/JCOG: Japan Clinical Oncology Group version of the Japanese translation of the Common Terminology Criteria for Adverse Events v5.0
MedDRA: Medical Dictionary for Regulatory Activities
MedDRA/J: Japanese version of the Medical Dictionary for Regulatory Activities
RSV: Respiratory syncytial virus
